# Secretion of intelectin-1 from malignant pleural mesothelioma into pleural effusion

**DOI:** 10.1038/sj.bjc.6605786

**Published:** 2010-07-13

**Authors:** S Tsuji, Y Tsuura, T Morohoshi, T Shinohara, F Oshita, K Yamada, Y Kameda, T Ohtsu, Y Nakamura, Y Miyagi

**Affiliations:** 1 Division of Cancer Therapy, Kanagawa Cancer Center Research Institute, 1-1-2 Nakao, Asahi-ku, Yokohama-shi, Kanagawa 241-0815, Japan; 2 Division of Pathology, Yokosuka-Kyosai Hospital, 1-16 Yonegahama-dori, Yokosuka-shi, Kanagawa 238-8558, Japan; 3 Division of General Thoracic Surgery, Yokosuka-Kyosai Hospital, 1-16 Yonegahama-dori, Yokosuka-shi, Kanagawa 238-8558, Japan; 4 Department of Clinical Research, Kochi National Hospital, 1-2-25 Asakura-nishimachi, Kochi-shi, Kochi 780-8077, Japan; 5 Department of Thoracic Oncology, Kanagawa Cancer Center, 1-1-2 Nakao, Asahi-ku, Yokohama-shi, Kanagawa 241-0815, Japan; 6 Department of Pathology, Kanagawa Cancer Center, 1-1-2 Nakao, Asahi-ku, Yokohama-shi, Kanagawa 241-0815, Japan; 7 Division of Molecular Pathology and Genetics, Kanagawa Cancer Center Research Institute, 1-1-2 Nakao, Asahi-ku, Yokohama-shi, Kanagawa 241-0815, Japan

**Keywords:** diagnosis, intelectin, mesothelial cells, mesothelioma, pleural effusion

## Abstract

**Background:**

Malignant pleural mesothelioma (MPM) is a rare but fatal tumour. Although most MPM patients show pleural effusion at even the early stage, it is hard to diagnose as MPM at the early stage because a sensitive and reliable diagnostic marker for MPM has not been found in plasma or pleural effusion.

**Methods:**

In this study, we investigated whether intelectin-1 was specifically contained in MPM cells and the pleural effusion of MPM patient by immunohistochemistry, western blotting, and enzyme-linked immunosorbent assay.

**Results:**

Malignant pleural mesothelioma cell lines, but not lung adenocarcinoma cell lines, secreted intelectin-1. In immunohistochemistry, epithelioid-type MPMs, but neither pleura-invading lung adenocarcinomas nor reactive mesothelial cells near the lung adenocarcinomas, were stained with anti-intelectin antibodies. Pleural effusion of MPM patients contained a higher concentration of intelectin-1 than that of lung cancer patients.

**Conclusion:**

These results suggest that detection of intelectin-1 may be useful for a differential diagnosis of epithelioid-type MPM in immunohistochemistry and that a high concentration of intelectin-1 in pleural effusion can be used as a new marker for clinical diagnosis of MPM.

Malignant pleural mesothelioma (MPM) is a rare but fatal tumour. Prognosis of MPM patients is very poor. The median survival is only 9–12 months after chemotherapy or radical surgery (Chailleux *et al*, 1988; Ruffie *et al*, 1989). Extrapleural pneumonectomy that is followed by chemotherapy and radiotherapy extends the median survival to 19 months (Sugarbaker *et al*, 1999). Recently, it was reported that patients who completed neoadjuvant pemetrexed plus cisplatin followed by extrapleural pneumonectomy and radiation had a median survival of 29.1 months and a 2-year survival rate of 61.2% (Krug *et al*, 2009). However, the surgery is generally applicable to only some MPM patients at the early stage.

It is hard to find MPM at the early stage for many reasons: it often progresses to an advanced stage without specific symptoms; radiographical diagnosis of MPM is difficult; and a sensitive and reliable diagnostic marker for MPM has not been found. As MPM is correlated with previous exposure to asbestos (Robinson and Lake, 2005), the number of MPM victims is increasing relative to the amount of asbestos used in the environment and would likely reach its peak between 2015 and 2020 in Western Europe (Peto *et al*, 1999) or in 2030 in Japan (Murayama *et al*, 2006). Thus, diagnosis of MPM at the early stage is urgently required.

Although most MPM patients show pleural effusion at even the early stage, a sensitive diagnostic marker for MPM has not been found in the pleural effusion. It is often difficult on cytological examination of the pleural effusion to even discriminate MPM cells from invasive lung adenocarcinomas or reactive mesothelial cells (Kitazume *et al*, 2000). For the differential diagnosis of MPM, some immunocytochemical tests for cells in the pleural effusion or some immunohistochemical tests for surgical pathologic specimens are generally required. Calretinin, cytokeratin 5/6, glucose transporter 1, mesothelin, and Wilm's tumour gene product WT-1 have been reported as immunohistochemical positive markers for epithelioid-type mesothelioma, which accounts for 50–80% of MPM cases (Ordóñez, 2003; Kato *et al*, 2007). However, these proteins are cytoplasmic or membrane-binding proteins and are not predominantly secreted into the pleural effusion. Therefore, these proteins undigested are difficult to detect in the pleural effusion and could not be used as a diagnostic marker for MPM in the pleural effusion.

Overexpression of intelectin-1 mRNA in MPM cells has been reported (Wali *et al*, 2005). Human intelectin-1 is a secretory trimeric protein (Tsuji *et al*, 2007) and a host defence lectin that binds to bacterial galactofuranose (Tsuji *et al*, 2009). mRNA of intelectin-1 is expressed primarily in the heart or intestine (Tsuji *et al*, 2001). Human intelectin-1 protein has been found in plasma of normal healthy adults (Tsuji *et al*, 2009) and has not been reported in other body fluids.

In this study, we analysed intelectin-1 produced by MPM cells and measured intelectin-1 concentrations in pleural effusions or plasmas of MPM patients. Epithelioid-type MPM cell lines, but not lung adenocarcinoma cell lines, secreted trimeric intelectin-1. In immunohistochemistry, epithelioid-type MPMs, but neither pleura-invading lung adenocarcinomas nor reactive mesothelial cells near the lung adenocarcinomas, were stained with anti-intelectin antibodies. Pleural effusions of MPM patients contained higher concentrations of intelectin-1 than those of lung cancer patients and tuberculosis patients. In the pleural effusion, there was no correlation between intelectin-1 and hyaluronic acid. These results suggest that intelectin-1 in the pleural effusion may be a proper diagnostic marker for MPM.

## Materials and methods

All experimental protocols were approved by the ethical committees of Kanagawa Cancer Center, Yokosuka-Kyosai Hospital, and Kochi National Hospital. A written informed consent for this study was obtained from each patient.

### Antibodies and intelectins

Recombinant intelectins and affinity-purified rabbit anti-intelectin polyclonal antibody (pAb) were prepared as described previously (Tsuji *et al*, 2007). cDNA encoding the open reading frame of human intelectin-2 was cloned from placental RNA by reverse transcription–polymerase chain reaction (RT–PCR). Anti-human intelectin-1 hybridomas were cloned from mouse myeloma cell lines fused with lymph node cells of human intelectin-1-immunised mice.

### Cell lines

Human MPM cell lines, ACC-MESO-1 (RCB2292) and ACC-MESO-4 (RCB2293) (Usami *et al*, 2006); a human lung adenocarcinoma cell line, A549 (RCB0098); and a human colon adenocarcinoma cell line, Caco-2 (RCB0988), were obtained from RIKEN Cell Bank (Tsukuba, Japan). Three human lung adenocarcinoma cell lines, ABC-1 (JCRB0815), RERF-LC-Ad2 (JCRB1021), and PC-3 (JCRB0077), were obtained from JCRB Cell Bank (Osaka, Japan). Four human MPM cell lines, NCI-H28 (CRL-5820), NCI-H2052 (CRL-5915), NCI-H2452 (CRL-5946), and MSTO-211H (CRL-2081), and a human prostate adenocarcinoma cell line, DU145 (HTB-81), were obtained from ATCC (Manassas, VA, USA). The human MPM cell lines – MEYK2 and MEYK4 – were established in this study from cells in pleural effusions of Japanese MPM patients, Y2 and Y4 (Table 1), respectively.

### Plasma and pleural effusion

Clinical information of the MPM samples is summarised in Table 1. Plasma and pleural effusion were heparinised, collected as centrifuged supernatants, and then stored at −80 °C. The MPM samples were obtained from Kanagawa Cancer Center or Yokosuka-Kyosai Hospital. Pleural effusions of non-MPM patients – 9 lung adenocarcinomas, 2 lung small cell carcinomas, 1 lung large cell carcinoma, 3 tuberculoses, and 10 bacterial pleuropneumonias – were obtained from the Department of Clinical Research, Kochi National Hospital. Normal healthy plasma was obtained from 19 healthy volunteers (9 men and 10 women; age range 25–69 years).

### Western blotting

Plasma (0.5 *μ*l), pleural effusion (0.5 *μ*l), or galactose-Sepharose-purified intelectin-1 from culture supernatant (2.5 ml) was resolved by 9% SDS–PAGE under non-reducing conditions and transferred to a polyvinylidene difluoride membrane (Immobilon-P; Millipore, Billerica, MA, USA). The membrane was blocked with 5% non-fat milk and then treated with affinity-purified anti-intelectin pAb or mAb. After washing, the membrane was treated with horseradish peroxidase-conjugated donkey anti-rabbit IgG (GE Healthcare UK Ltd, Buckinghamshire, UK) or horseradish peroxidase-conjugated sheep anti-mouse IgG (GE Healthcare UK Ltd) and developed with ECL advance (GE Healthcare UK Ltd). The membrane was reprobed by incubating with 6 M guanidine HCl (pH 6.8), washed with water, and re-blocked with 5% non-fat milk.

### RT–PCR

Total RNA was purified with TRIzol Reagent (Invitrogen, Carlsbad, CA, USA) from ACC-MESO-1 or ACC-MESO-4. RNA (1 *μ*g) was reverse transcribed and amplified as cDNA with the OneStep RT–PCR kit (Qiagen GmbH, Hilden, Germany) (0.6 *μ*M primers (5′-GTGGAGGGAGGGAGTGAAGGAG-3′ and 5′-GAGTCAATATGATTTATTGTTTTCTCTTCTG-3′) at 94 °C for 30 s, 56 °C for 30 s, and 72 °C for 1 min). PCR products were resolved by 1.5% agarose gel electrophoresis and stained by ethidium bromide. PCR products were also sequenced directly.

### Immunohistochemistry

Specimens were obtained from the Division of Pathology in Kanagawa Cancer Center or Yokosuka-Kyosai Hospital. Pleural biopsy-resected tissue was fixed with formalin and prepared as paraffin-embedded thin-sliced sections. The section on glass slide was deparaffinised and rehydrated with xylene and ethanol and then heated at 121 °C for 5 min with an antigen retrieval buffer, 10 mM Tris buffer (pH 9.0) containing 1 mM ethylenediaminetetraacetic acid (EDTA). Endogenous peroxidase activity was eliminated by incubating for 10 min with 3% hydrogen peroxide. After washing with phosphate-buffered saline, the section was treated for 1 h with 1.5 *μ*g ml^−1^ of affinity-purified anti-intelectin pAb or mAb, 15:3G9. After washing with 20 mM Tris-buffered saline (pH 7.6) containing 0.05% Tween 20 (TBST), immunoreactivity was visualised by using Histofine Simple Stain MAX-PO (R) or MAX-PO (M) (Nichirei Co., Tokyo, Japan) and 3,3′-diaminobenzidine according to the manufacturer's instructions. The section was finally counterstained with haematoxylin, dehydrated, and mounted with Malinol medium (Muto Pure Chemicals Co., Tokyo, Japan). Calretinin or thyroid transcription factor-1 (TTF-1) was stained with anti-calretinin (rabbit anti-calretinin mAb (SP13), Nichirei Co.) or anti-TTF-1 (mouse anti-TTF-1 mAb (8G7G3/1), DAKO Japan Inc., Tokyo, Japan) according to the manufacturer's instructions, respectively.

### Enzyme-linked immunosorbent assay (ELISA)

The concentration of intelectin-1 was measured by sandwich ELISA. Plasma, pleural effusion, or culture supernatant was diluted 100 times, 100–1000 times, or 20 times with TBST plus 0.2% bovine serum albumin (BSA), respectively. The intelectin-1 was captured by affinity-purified anti-intelectin pAb immobilised on 96-well plates and washed three times with TBST. The plates were incubated with TBST plus 0.2% BSA containing mAb. After washing, the plates were incubated with TBST plus 0.2% BSA containing horseradish peroxidase-conjugated sheep anti-mouse IgG (GE Healthcare UK Ltd), washed, and developed with 3,3′,5,5′-tetramethylbenzidine (Thermo Fisher Scientific Inc., Rockford, IL, USA), and then the absorbance at 450 nm was determined. Recombinant human intelectin-1 for a standard was purified from human intelectin-1-transfected RK-13 cells using galactose-Sepharose (Tsuji *et al*, 2001). The concentration of intelectin-1 standard was estimated on the basis of the absorbance at 280 nm and the predicted extinction coefficient (Mach *et al*, 1992) of human intelectin-1.

The concentration of hyaluronic acid in pleural effusion was measured using Hyaluronan Assay Kit (Seikagaku Biobusiness Co., Tokyo, Japan).

## Results

### Antibodies against human intelectins

**Figure 1:**
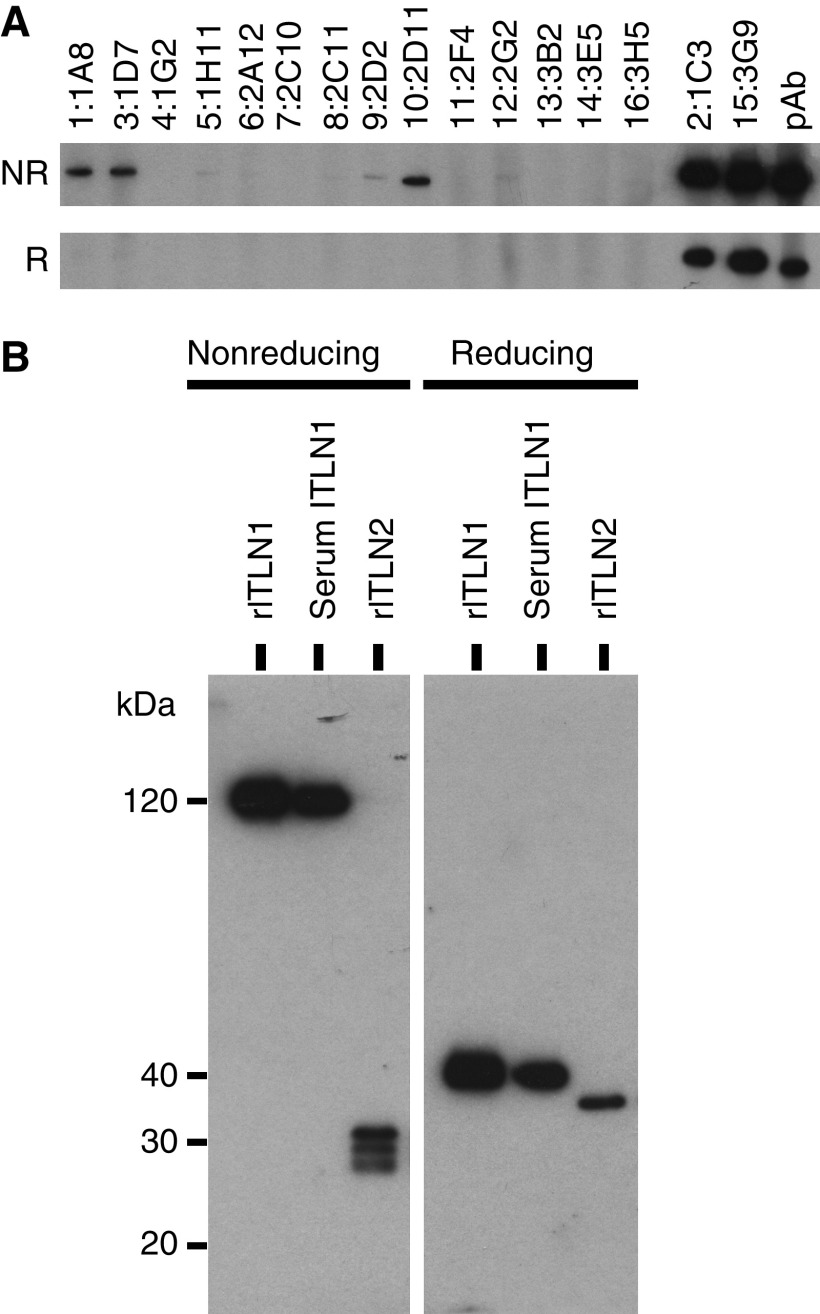
Western blotting using anti-intelectin antibodies. (**A**) Recombinant human intelectin-1 (5 ng per lane) was resolved by SDS–PAGE under the non-reducing (NR) or the reducing (R) conditions and detected by western blotting using mAb or affinity-purified anti-intelectin pAb. (**B**) Recombinant intelectin-1 (rITLN1) and recombinant intelectin-2 (rITLN2) were purified with galactose-Sepharose from culture supernatant of transiently intelectin-transfected RK-13 cells. Serum intelectin-1 (Serum ITLN1) was purified from the serum of healthy human donors. The samples were detected by western blotting using anti-intelectin pAb, as described in the Materials and Methods.

The antibodies used in this study are summarised in Figure 1 and Supplementary Table 1. The mAbs – 1:1A8, 2:1C3, 3:1D7, 10:2D11, and 15:3G9 – that recognised the N-terminus of human intelectin-1 (Supplementary Table 1) detected non-reducing intelectin-1 on western blotting (Figure 1A). Two mAbs, 5:1H11 and 9:2D2, that bound to the C-terminus of intelectin-1 (Supplementary Table 1) weakly bound to non-reducing intelectin-1 on western blotting (Figure 1A). These mAbs, except for 12:2G2, did not recognise intelectin-2, which is another homologue in humans (Supplementary Table 1). Affinity-purified anti-intelectin pAb bound to both the N-terminus and the C-terminus of intelectin-1 or intelectin-2 because the pAb detected reducing or non-reducing intelectin-1 and intelectin-2 (Figure 1B) and captured these intelectins (Supplementary Table 1).

Human intelectin-1 was a disulphide-linked trimer with glycosylation, as described in a previous report (Tsuji *et al*, 2007). On the other hand, intelectin-2 migrated to molecular size corresponding to a monomer under the non-reducing conditions (Figure 1B). A single 34 kDa band of intelectin-2, which was equivalent to molecular weight without glycosylation, was shown under the reducing conditions, indicating that three bands of non-reducing intelectin-2 were derived from unequal disulphide-linked formation of intelectin-2 (Figure 1B). These results suggest that both intelectin-1 and intelectin-2 are detectable and distinguishable by western blotting using pAb.

### Secretion of intelectin-1 from mesothelioma cells

**Figure 2:**
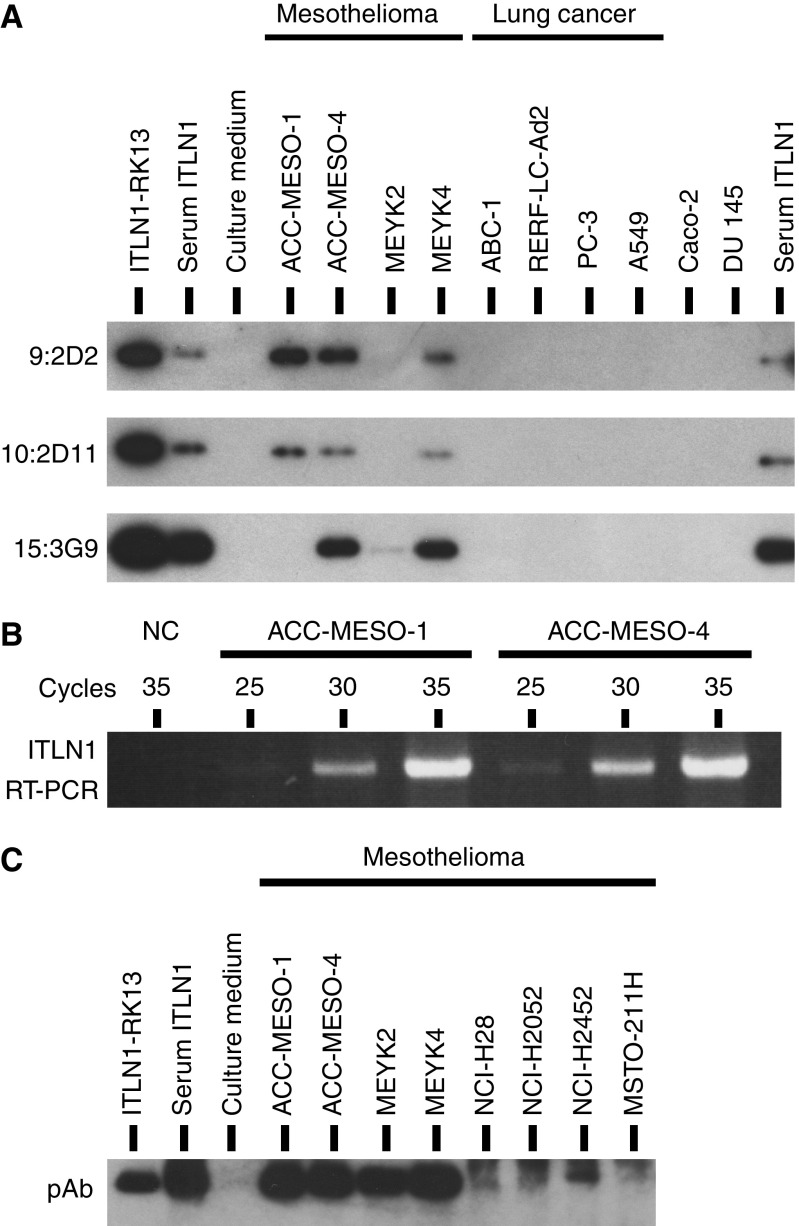
Production of intelectin-1 in MPM cell lines. (**A**) Intelectin-1 was purified with galactose-Sepharose from 3 days of culture supernatant containing 10% fetal bovine serum and was detected as a single 120 kDa band by non-reducing western blotting. Intelectin-1-transfected RK-13 cells (ITLN-RK13) were cultured in medium containing 5% fetal bovine serum. Results were obtained by reprobing and reblotting of an identical membrane as described in the Materials and Methods. Serum intelectin-1 (Serum ITLN1) was prepared from the serum of healthy human donor. (**B**) Intelectin-1 mRNAs of two MPM cell lines were compared using semiquantitative RT–PCR. A sample without RNA was used as a negative control (NC). (**C**) Intelectin-1 was purified with galactose-Sepharose from 5 days of culture supernatant containing 10% fetal bovine serum and was detected by non-reducing western blotting using affinity-purified anti-intelectin pAb.

Anti-intelectin-1 mAbs, 9:2D2 or 10:2D11, recognised an epitope in the C-terminus or N-terminus of human intelectin-1, respectively (Supplementary Table 1). As shown in Figure 2A, three human mesothelioma cell lines – ACC-MESO-1, ACC-MESO-4, and MEYK4 – secreted 120 kDa trimeric intelectin-1 recognised by these mAbs into the culture supernatant. In contrast, four lung adenocarcinomas (ABC-1, RERF-LC-Ad-2, PC-3, and A549), a colon adenocarcinoma (Caco-2), and a prostate adenocarcinoma (DU145) did not express intelectin-1. A mAb, 15:3G9, recognised an epitope in the N-terminus of human intelectin-1 (Supplementary Table 1) and sensitively detected reducing or non-reducing intelectin-1 on western blotting (Figure 1A). A small amount of intelectin-1 secreted from MEYK2 was detected by 15:3G9 (Figure 2A). The intelectin-1 of ACC-MESO-4 or MEYK4 was also detected by this mAb, but intelectin-1 of ACC-MESO-1 was not. Intelectin-1 of non-mesothelioma cell lines was not detected by 15:3G9. There was no difference between the intelectin-1 mRNA of ACC-MESO-1 and that of ACC-MESO-4; both cell lines transcribed a similar amount of intelectin-1 mRNA (Figure 2B) and each intelectin-1 had the same single-nucleotide polymorphisms: A48G (silent mutation), T258C (silent mutation), and A326T (D109V). Mesothelioma cell lines derived from epithelioid-type MPM – ACC-MESO-1, ACC-MESO-4, MEYK2, MEYK4, NCI-H28, NCI-H2052, and NCI-2452 – secreted intelectin-1 detected by anti-intelectin-1 pAb, yet MSTO-211H derived from lung-metastatic site of biphasic MPM did not secrete intelectin-1 (Figure 2C). These results suggest that epithelioid-like mesothelioma cell lines, but not the non-mesothelioma lines, secrete trimeric intelectin-1. In addition, the results also indicate that the intelectin-1 of ACC-MESO-1 lacks an epitope of 15:3G9, although the coding sequence of *itln-1* gene of ACC-MESO-1 is the same as that of ACC-MESO-4.

### Immunohistochemistry of MPM

**Figure 3:**
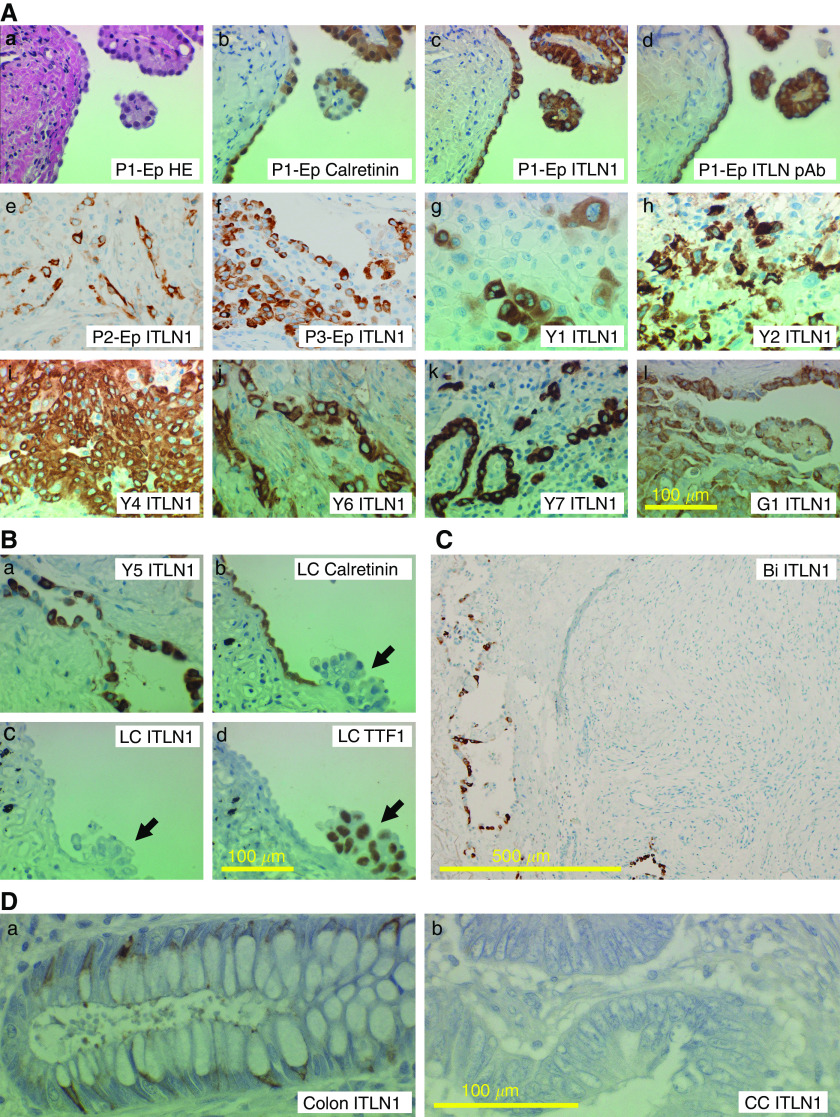
Immunohistochemistry of intelectin-1 in MPM. Specimens of pleural biopsy were analysed by immunohistochemistry. Patients Y1, Y2, Y4, Y5, Y6, Y7, and G1 are identical to the ones in Table 1, respectively. Photographs were taken with a × 10 (panel **C**) or × 40 (others) objective lens. A scale bar is shown in the bottom side of each panel, representatively. (**Aa**) Haematoxylin–eosin (HE) staining of a specimen of an epithelioid-type MPM patient (P1-Ep). (**Ab**) Calretinin staining of MPM in the same specimen. (**Ac**) Intelectin-1 (ITLN1) staining of MPM in the same specimen with 15 : 3G9. (**Ad**) ITLN1 staining of MPM in the same specimen with anti-intelectin pAb. (**Ae**–**Al**) ITLN1 staining of MPM in eight epithelioid-type MPM patients (P2-Ep, P3-Ep, Y1, Y2, Y4, Y6, Y7, or G1) with 15 : 3G9 (**Ae**–**Ak**) or anti-intelectin pAb (**Al**). (**Ba**) ITLN1 staining of pleural surface MPM in an epithelioid-type MPM patient (Y5). (**Bb**) Calretinin staining of reactive mesothelial cells on a pleura invaded by lung adenocarcinoma in a lung cancer patient (LC). (**Bc**) No staining of the pleural cells in the same specimen with 15:3G9. (**Bd**) TTF-1 staining of pleura-invading lung adenocarcinoma in the same specimen. The arrows indicate the lung adenocarcinomas invading the pleura. (**C**) ITLN1 staining in a biphasic-type MPM patient (Bi). ITLN1 was stained with 15 : 3G9 in epithelioid-like MPMs in the left side, but not sarcomatoid-like MPMs in the centre and right side. (**Da**) ITLN1 staining of normal colonic goblet cells. (**Db**) No staining of colon adenocarcinoma in the identical patient (CC) with anti-intelectin pAb.

The results of MPM immunohistochemistry are shown in Figure 3. Intelectin-1 was detected in the cytoplasm but not in the nucleus (Figure 3). Epithelioid-type MPMs were immunostained with anti-intelectin antibodies, 15:3G9 or pAb, as well as antibody against calretinin, a typical positive marker for epithelioid-type mesothelioma (Figure 3Aa–Ad). All tested epithelioid-type MPMs were stained with anti-intelectin antibodies; well-differentiated epithelioid-type mesotheliomas near a pleura surface tended to express intelectin-1 (Figure 3A), whereas a small number of poorly differentiated epithelioid-type mesothelioma produced intelectin-1 (Figure 3Ag). Pleura surface mesothelioma cells expressed both calretinin (Figure 3Ab) and intelectin-1 (Figure 3Ac and Ba); in contrast, reactive mesothelial cells on a lung adenocarcinoma-invaded pleura expressed calretinin (Figure 3Bb) but not intelectin-1 (Figure 3Bc). The pleura-invading lung adenocarcinoma, which expressed TTF-1 (Figure 3Bd, arrow), was not stained with both anti-calretinin (Figure 3Bb, arrow) and anti-intelectin (Figure 3Bc, arrow). In specimens of eight pleuritis patients with lung adenocarcinoma, no cell expressed intelectin-1 (data not shown). In a biphasic-type MPM patient, epithelioid-like mesothelioma cells (Figure 3C, left side) but not sarcomatoid-like cells (Figure 3C, right side) expressed intelectin-1. These results suggest that epithelioid-type MPM specifically expresses intelectin-1 protein. The detection of intelectin-1 expression in a pleural biopsy sample may be useful for differential diagnosis of epithelioid-type MPM, because invasive lung adenocarcinomas and calretinin-positive reactive mesothelial cells, which often cause a difficult diagnosis of MPM, did not express intelectin-1. Because intelectin-1 mRNA is expressed in normal intestines (Tsuji *et al*, 2001), we also investigated expression of intelectin-1 in colon cancer. Intelectin-1 was detected in the cytoplasm of normal colonic goblet cells (Figure 3Da) but not that of colon adenocarcinomas (Figure 3Db). In seven patients, colon carcinomas did not express intelectin-1 (data not shown). Thus, colon cancer would not increase the amount of intelectin-1 in a body.

### Intelectin-1 in plasma and pleural effusion

**Figure 4:**
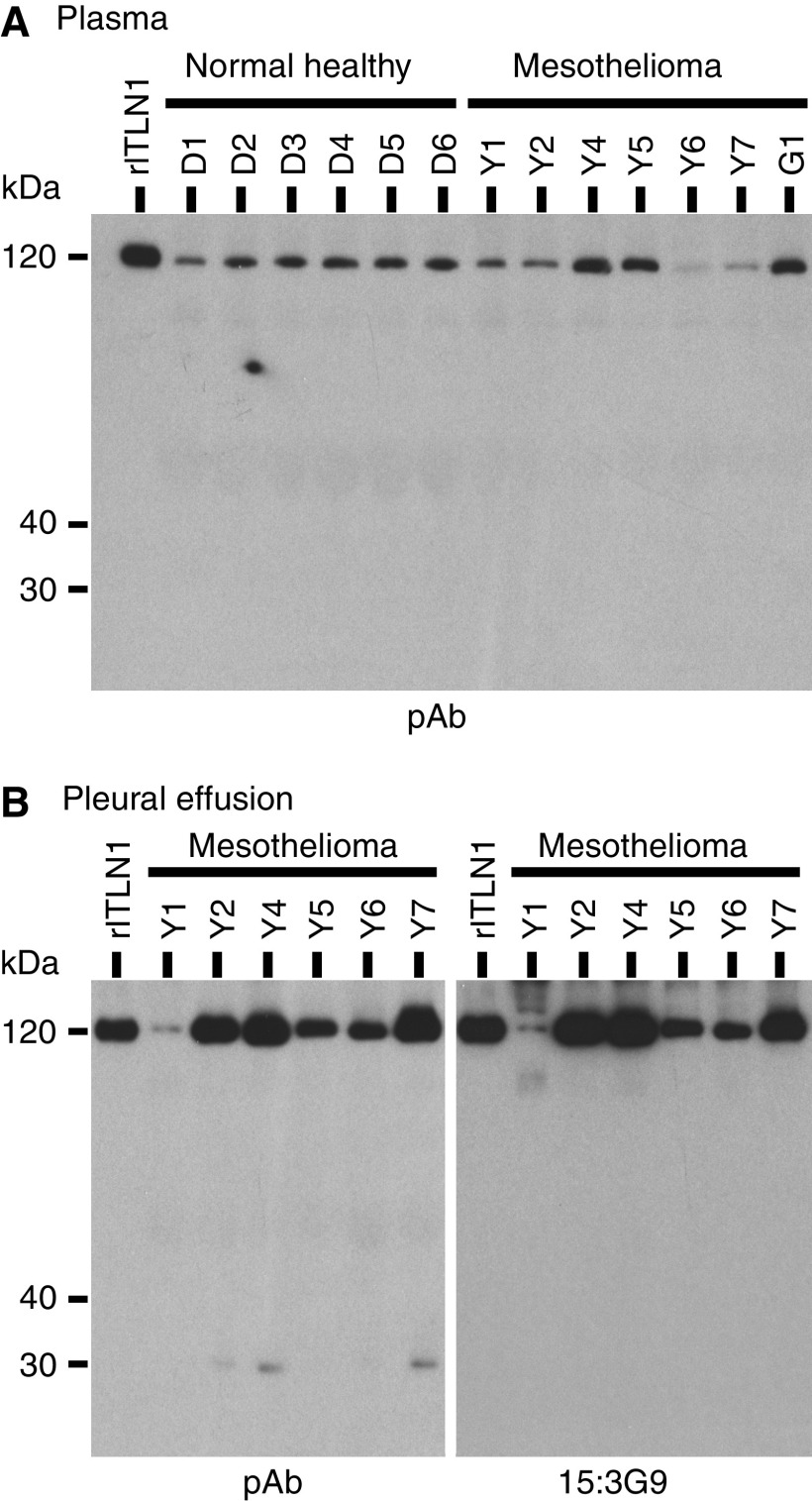
Western blotting of intelectin-1 in pleural effusion and plasma. Recombinant intelectin-1 (rITLN1) is used as a positive control. Mesothelioma patients of Y1, Y2, Y4, Y5, Y6, Y7, and G1 are identical to the ones in Table 1, respectively. (**A**) Plasma (0.5 *μ*l) was analysed by non-reducing western blotting using affinity-purified anti-intelectin pAb. (**B**) Pleural effusion (0.5 *μ*l) was analysed by non-reducing western blotting using affinity-purified anti-intelectin pAb or 15:3G9. Results were obtained by reprobing and reblotting of an identical membrane as described in the Materials and Methods.

Trimeric intelectin-1 (120 kDa) was detected in plasma (Figure 4A) and pleural effusion (Figure 4B) of all MPM patients on western blotting. As shown in Figure 4, most pleural effusions contained larger amounts of intelectin-1 than plasma. The amount of intelectin-1 in the plasma of MPM patients was inconsistent and did not correlate with that of the pleural effusion. There was no obvious shift in the electrophoretic mobility of trimeric intelectin-1 of MPM patients compared with plasma intelectin-1 of normal healthy donors. A band of monomeric intelectin (30 kDa) was not detected with pAb in plasma (Figure 4A), whereas it was detected in pleural effusions of some MPM patients (Figure 4B, Y2, Y4, and Y7) with pAb but not 15:3G9. These results suggested that the pleural effusions of some MPM patients contained small amounts of intelectin-2 with intelectin-1.

**Figure 5:**
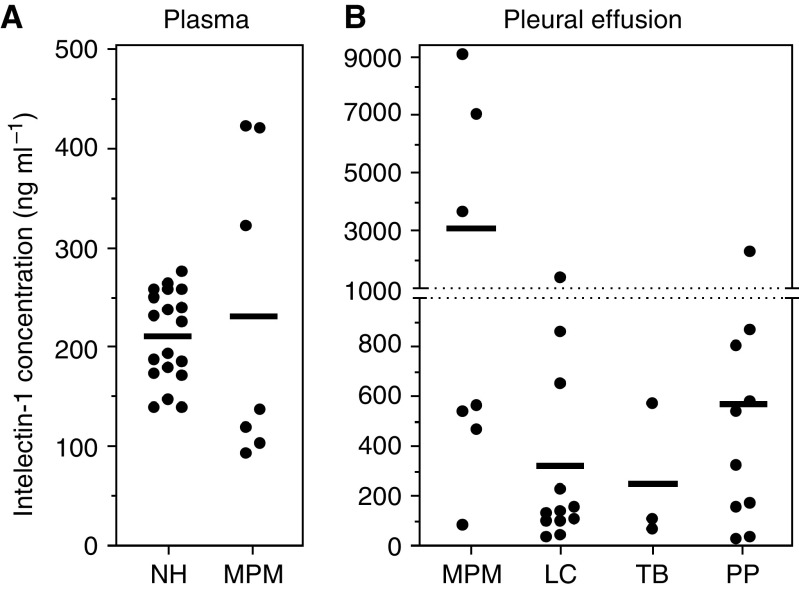
Concentration of intelectin-1 in pleural effusion and plasma. Intelectin-1 concentration was measured by sandwich ELISA as described in the Materials and Methods. Values represent the mean of duplicate determinations. The bold horizontal bar is the mean value of each sample. (**A**) Intelectin-1 concentration in heparinised plasmas of normal healthy donors (NH) or MPM patients (MPM). (**B**) Intelectin-1 concentration in heparinised pleural effusions of patients with MPM (MPM), lung cancer (LC), tuberculosis (TB), and pleuropneumonia (PP). Lung cancer contains 9 adenocarcinomas, 2 small cell carcinomas (intelectin-1 concentration, 1359 and 98 ng ml^−1^), and 1 large cell carcinoma (intelectin-1 concentration, 222 ng ml^−1^). Differences in mean values were evaluated by permutation tests. The *P*-value between MPM and LC was 0.0117. The *P*-value between MPM and PP was 0.0306.

The concentration of intelectin-1 in plasmas and pleural effusions is shown in Figure 5 and Table 1. The plasmas of three MPM patients (Y4, Y5, and G1) contained intelectin-1 at higher concentrations than those of normal healthy donors (Figure 5A and Table 1). However, there was little difference between the mean concentration of plasma intelectin-1 in MPM patients and that in healthy donors (Figure 5A). On clinical diagnosis, it is important that the pleural effusion of MPM is distinguished from that of lung cancer or tuberculosis. The pleural effusions of epithelioid-type MPM patients were apt to contain higher concentrations of intelectin-1 than those of lung cancer or tuberculosis patients (Figure 5B and Table 1). Half of the pleural effusions of MPM patients (Y2, Y4, and Y7), but not patients with other diseases, contained >3000 ng ml^−1^ of intelectin-1. Most pleural effusions of patients with other diseases contained <1000 ng ml^−1^ of intelectin-1 (Figure 5B). A high concentration of hyaluronic acid (>100 *μ*g ml^−1^) in pleural effusion is a reliable diagnostic marker for MPM (Petterson *et al*, 1988; Welker *et al*, 2007). The pleural effusions of half of the MPM patients (Y2, Y6, Y7, and G2) contained >100 *μ*g ml^−1^ of hyaluronic acid (Table 1). There was no correlation between intelectin-1 concentration and hyaluronic acid concentration in the pleural effusions of MPM patients (Table 1) or other diseases (data not shown). These results suggested that a high concentration of intelectin-1 (>3000 ng ml^−1^) in pleural effusion can be used as another marker for the clinical diagnosis of MPM.

## Discussion

Hyaluronic acid (Petterson *et al*, 1988; Welker *et al*, 2007) and soluble mesothelin (Maeda and Hino, 2006) have been known as diagnostic markers for MPM in pleural effusion and plasma, respectively. High concentration of hyaluronic acid (>100 *μ*g ml^−1^) in the pleural effusion is a reliable marker for MPM (Petterson *et al*, 1988; Welker *et al*, 2007). However, in many clinical cases, the only measurement of hyaluronic acid is insufficient for definite diagnosis of MPM because many MPMs have pleural effusions with less hyaluronic acid. Mesothelin is a glycosylphosphatidylinositol-anchored membrane protein expressed on mesothelial cells (Maeda and Hino, 2006). The concentration of soluble mesothelin antigen rises in the serum of epithelioid-type MPM patients (Robinson *et al*, 2003). Although a truncating variant and proteolytic peptides have been reported as soluble mesothelin, most soluble mesothelin would be the digested mesothelin shed from the cell surface rather than the truncating variant (Segawa *et al*, 2008). The proteolytic sensitivity of mesothelin may be an obstacle to reproducibly quantifying mesothelin. Therefore, mesothelin would be an unsuitable diagnostic marker in an accumulative pleural effusion. In this study, we showed that mesothelioma cells specifically secreted intelectin-1 and that the pleural effusions of MPM patients contained large amounts of intelectin-1. Intelectin-1 was not proteolysed at 37 °C for 24 h in plasma or pleural effusion (Supplementary Table 2). Thus, intelectin-1 in pleural effusion could be used as a specific and stable diagnostic marker for MPM.

In pleural biopsy-resected tissue, epithelioid-type MPM – but neither pleura-invading lung adenocarcinomas nor reactive mesothelial cells near the lung adenocarcinomas – specifically expressed intelectin-1 protein (Figure 3). In a biphasic-type MPM, only epithelioid-like mesothelioma cells expressed intelectin-1 (Figure 3C). Intelectin-1 expression in epithelioid-type MPMs near a pleura surface would be a suitable effect for the intelectin-1 secreted into the pleural cavity. It is often difficult to discriminate noninvasive MPM cells on a pleura surface from pleura-invading lung adenocarcinoma or reactive mesothelial cells because these cells resemble each other in morphology and/or immunohistology (Kitazume *et al*, 2000). For example, calretinin was detected in both reactive mesothelial cells and epithelioid-type MPM cells (Figure 3Bb and Ab). On the other hand, intelectin-1 was not expressed in either reactive mesothelial cells or pleura-invading lung adenocarcinoma (Figure 3Bc). Although additional investigation in reactive mesothelial cells in other diseases than carcinomatous pleuritis is required, the immunohistochemistry with anti-intelectin-1 may be useful for differential diagnosis of epithelioid-type MPM.

Intelectin expression increases during gastrointestinal infection (Datta *et al*, 2005; French *et al*, 2008). Human intestinal intelectin-1 was produced from goblet cells and secreted into mucus (Figure 3Da). In opposition to MPM, colon adenocarcinoma cells lost expression of intelectin-1 (Figure 3Db), and the Caco-2 colon adenocarcinoma cell lines did not secrete intelectin-1 (Figure 2A). Thus, the amount of intelectin-1 in a body would not increase because of colon cancer. It was also reported that intelectin-1 was related to chronic obstructive pulmonary disease (Carolan *et al*, 2008) and asthma (Kuperman *et al*, 2005). Pleural effusion with pleuropneumonia tended to contain a little more intelectin-1 than that with lung cancer (Figure 5B). Thus, intelectin-1 may be induced in mesothelial cells by an inflammatory stimulation, such as Th2 cytokine, and function as a host defence protein in relation to a respiratory inflammation. Asbestos induces pleural inflammation (Kroegel and Antony, 1997). Furthermore, it was reported that asbestos directly induced intelectin-1-expression in mesothelial cells (Wali *et al*, 2005). To analyse the neoplastic transformation mechanism of mesothelial cells expressing intelectin-1, it may be useful to investigate further what stimulation, including asbestos, induces expression of intelectin-1 in mesothelial cells.

Intelectin-1 of ACC-MESO-1 was not identified by western blotting using 15:3G9 despite its detection with other mAbs against intelectin-1 (Figure 2A). This result suggests that intelectin-1 of ACC-MESO-1 lacks an epitope of 15:3G9 in the N-terminus of serum intelectin-1. The intelectin-1 of ACC-MESO-1 had neither single-nucleotide polymorphisms nor obvious shift in electrophoretic mobility in comparison with the intelectin-1 of ACC-MESO-4 that was detectable with 15:3G9 (see Results section and Figure 2A). Furthermore, an unglycosylated mutant of intelectin-1 was also recognised with 15:3G9 (Supplementary Table 1). Thus, intelectin-1 of ACC-MESO-1 would have neither long deletion of the N-terminus nor deficiency of *N*-linked glycosylation. These results suggest that intelectin-1 of ACC-MESO-1 does not have a 15:3G9-recognised post-translational modification of normal serum intelectin-1. Shortly, we would analyse the epitope of 15:3G9 and the post-translational modification of intelectin-1 because the rate of this modification might vary with a disease such as MPM.

## Figures and Tables

**Table 1:** Clinical data of MPM patients and intelectin-1 concentration in their body fluids

					**Plasma**	**Pleural effusion**	
**ID**	**Sex/age**	**Type**	**Stage**	**Asbestos exposure**	**Intelectin-1 (ng ml^−1^)**	**Intelectin-1 (ng ml^−1^)**	**Hyaluronic acid (*μ*g ml^−1^)**
Y1	M/76	Epithelioid (PD)	T3N2M0	+	135.8	83.6	27.0
Y2	M/74	Epithelioid	T1bN0M0	+	118.6	3609.0	811.6
Y4	M/65	Epithelioid	T3N0M0	+	420.0	9071.8	41.9
Y5	M/69	Epithelioid	T1-2N0M0	+	320.3	537.0	90.6
Y6	M/61	Epithelioid	T3		102.6	565.3	149.3
Y7	M/66	Epithelioid	T3		92.0	7000.3	237.8
G1	M/68	Epithelioid	T4N0M0	+	422.6	ND	ND
G2	M/60	ND	T4N0M0		ND	463.8	203.5

Abbreviations: MPM=malignant pleural mesothelioma; ND=not determined; PD=poorly differentiated.

The concentration of intelectin-1 or hyaluronic acid was measured by ELISA as described in the Materials and Methods. Values represent the mean of duplicate determinations.
