# Supplementary material for: Secretion of intelectin-1 from malignant pleural mesothelioma into pleural effusion
Source: Br J Cancer. 2010 Jul 13;103(4):517–23. doi: 10.1038/sj.bjc.6605786 (PMC2939784; doi:10.1038/sj.bjc.6605786)
Supplement: Supplementary Table S1 [file 6605786x1.doc]

**Supplementary Table S1. Summary of characteristics of anti-intelectin antibodies.**

| Name | Subclass |  | ELISA | |  | Sandwich ELISA (capture, pAb) | | | | | | | |
| --- | --- | --- | --- | --- | --- | --- | --- | --- | --- | --- | --- | --- | --- |
|  |  |  | BSA | ITLN1 |  | medium | ITLN1 | N-hITLN1 | C-hITLN1 | ITLN2 | ITLN1  +EDTA | monomeric  ITLN1 | unglycosylated  ITLN1 |
| 1:1A8 | mouse IgG3 |  | 0.033 | >2.0 |  | 0.057 | 0.089 | 0.111 | 0.053 | 0.063 | 0.183 | 0.154 | 0.095 |
| 2:1C3 | mouse IgG2a |  | 0.030 | >2.0 |  | 0.058 | 0.363 | 1.178 | 0.053 | 0.050 | 1.057 | 0.782 | 0.373 |
| 3:1D7 | mouse IgG2b |  | 0.049 | >2.0 |  | 0.100 | 0.111 | 0.090 | 0.080 | 0.099 | 0.108 | 0.093 | 0.098 |
| 10:2D11 | mouse IgG1 |  | 0.025 | >2.0 |  | 0.053 | 1.580 | 0.306 | 0.052 | 0.050 | 1.669 | 0.111 | 1.447 |
| 15:3G9 | mouse IgG2a |  | 0.029 | >2.0 |  | 0.061 | 0.584 | >2.0 | 0.067 | 0.067 | >2.0 | 1.222 | 0.538 |
|  |  |  |  |  |  |  |  |  |  |  |  |  |  |
| 5:1H11 | mouse IgG2a |  | 0.043 | 0.466 |  | 0.099 | >2.0 | 0.075 | >2.0 | 0.112 | >2.0 | 0.780 | >2.0 |
| 6:2A12 | mouse IgG1 |  | 0.062 | 0.275 |  | 0.076 | >2.0 | 0.052 | 0.701 | 0.065 | 1.745 | 0.446 | 1.729 |
| 9:2D2 | mouse IgG1 |  | 0.026 | 0.484 |  | 0.058 | >2.0 | 0.052 | >2.0 | 0.047 | >2.0 | 1.172 | >2.0 |
| 12:2G2 | mouse IgG1 |  | 0.031 | 0.224 |  | 0.053 | >2.0 | 0.052 | 1.188 | 0.131 | 1.803 | 0.478 | 1.731 |
|  |  |  |  |  |  |  |  |  |  |  |  |  |  |
| pAb | rabbit IgG |  | 0.050 | >2.0 |  |  |  |  |  |  |  |  |  |

Results are shown as the means of absorbance at 450 nm of duplicate determinations. Binding of mAb to intelectin-1 was estimated by an indirect ELISA for purified recombinant human intelectin-1 immobilized on a microtiter plate. An epitope of mAb was determined by sandwich ELISA using affinity-purified anti-intelectin-1 pAb as a capture. Intelectins were provided as recombinant proteins from a culture supernatant with human intelectin-1 (ITLN1), ITLN1 plus 10 mM EDTA (ITLN1 + EDTA), human intelectin-2 (ITLN2), a chimeric molecule consisting of the N-terminus of human intelectin-1 and the C-terminus of mouse intelectin-1 (N-hITLN1), a chimeric molecule consisting of the C-terminus of human intelectin-1 and the N-terminus of mouse intelectin-1 (C-hITLN1), C31,48S ITLN1 (monomeric ITLN1), or N163K ITLN1 (unglycosylated ITLN1). A culture medium without intelectin was used as a negative control. N-hITLN1, C-hITLN1, monomeric ITLN1, and unglycosylated ITLN1 were prepared as described previously (Tsuji S *et al.* (2007) *Glycobiology* **17:** 1045). The mAb subclass was determined by using an IsoStrip Mouse Monoclonal Antibody Isotyping Kit (Roche Diagnostics K. K., Tokyo, Japan).
