# Supplementary material for: Secretion of intelectin-1 from malignant pleural mesothelioma into pleural effusion
Source: Br J Cancer. 2010 Jul 13;103(4):517–23. doi: 10.1038/sj.bjc.6605786 (PMC2939784; doi:10.1038/sj.bjc.6605786)
Supplement: Supplementary Table S2 [file 6605786x2.doc]

Supplementary Table S2. Resistance of intelectin-1 to proteolysis in plasma and pleural effusion.

|  | Initial concentration of intelectin-1 | After incubation at 37ºC for 24 h |
| --- | --- | --- |
| Y4 plasma | 499.8 ± 15.3 | 543.8 ± 15.2 |
| Y4 pleural effusion | 8491.8 ± 274.4 | 9232.0 ± 175.3 |
| Y5 plasma | 362.7 ± 9.8 | 372.3 ± 6.8 |
| Y5 pleural effusion | 639.1 ± 12.1 | 662.2 ± 19.2 |

Results are shown as the means ± SEM of triplicate determinations. Plasmas and pleural effusions of the Y4 or Y5 patient were incubated at 37°C for 24 h. Samples were diluted and their intelectin-1 concentrations were measured by sandwich ELISA as described in *Materials and Methods*.
